# Effect of Health Literacy on Decision Delay in Patients With Acute Myocardial Infarction

**DOI:** 10.3389/fcvm.2021.754321

**Published:** 2021-11-30

**Authors:** Zhao-ya Fan, Yuan Yang, Ruo-yun Yin, Lei Tang, Fan Zhang

**Affiliations:** ^1^Research Center for Medicine and Social Development, Collaborative Innovation Center of Social Risks Governance in Health, School of Public Health and Management, Chongqing Medical University, Chongqing, China; ^2^Department of Cardiovascular Medicine, The First Affiliated Hospital of Chongqing Medical University, Chongqing, China

**Keywords:** acute myocardial infarction, decision delay, health literacy, patient decision making, pre-hospital delay

## Abstract

**Background:** Health literacy (HL) is a risk factor for adverse outcomes in patients with cardiovascular disease, and shorter pre-hospital delay time is crucial for successful treatment of acute myocardial infraction (AMI) patients. Most previous studies focused on the influencing factors of pre-hospital delay but ignore the essential contribution of decision delay.

**Aims:** Therefore, the purpose of this study was to explore the effect of HL on decision delay.

**Methods:** Continuously included AMI patients admitted to a grade A class three hospital in Chongqing. HL level was assessed using Brief Health Literacy Screen and categorized as adequate or inadequate. Mann-Whitney *U*-test and Chi-square test were used to compare the differences between groups, and binary logistic regression was used to analyze the association between HL and decision delay.

**Results:** A total of 217 AMI patients were enrolled in this study, including 166 males (76.5%) and 51 females (23.5%), with the median age was 68 years old; 135 (62.2%) patients had delayed decision-making while 82 (37.8%) did not; 157 (72.7%) patients had inadequate HL and 59 (27.3%) had adequate HL. The total HL score of non-delayed group was higher than that in delayed group (9.22 vs. 7.02, *P* < 0.000).

**Conclusion:** After adjusting for covariates, HL was significantly negatively associated with decision time. AMI patients with inadequate HL were more likely to delay seeking timely medical care.

## Introduction

Cardiovascular Diseases (CVDs) are important factors endangering people's health worldwide, mainly including coronary artery disease (CAD), hypertension, and chronic heart failure (HF) (1). The report showed that 42.6% of Americans who died from CVD were related to CAD (2). Although the prevalence and mortality of CAD have declined in recent years, it is still the leading cause of death in the United States (3). The prevalence of CVDs in China is also not optimistic. It is estimated that there are 330 million people suffering from CVDs, including 11 million cases of CAD and 5 million cases of HF. Two out of every five deaths are due to CVDs (4). Acute myocardial infarction (AMI) is myocardial necrosis caused by acute and persistent ischemia and hypoxia of coronary artery. According to the electrocardiogram, ST-segment elevation myocardial infraction (STEML) and non-ST segment elevation myocardial infraction (NSTEML) were classified (5). The improvement of short-term and long-term survival in patients with AMI is closely related to the reperfusion time (6). Reperfusion therapy can effectively avoid the expansion of myocardial infraction areas and reduce the mortality of patients. Every 10 min delay in blood flow recovery results in an additional 3.31 deaths in 100 patients treated with percutaneous coronary intervention (PCI) (7). Therefore, in order to reduce the morbidity and mortality of AMI, early and accurate identification of related symptoms and rapid recanalization of occluded blood vessels are essential.

Delays are common in AMI patients in the process of seeking medical treatment. Treatment seeking delay refers to the special behavior that an individual delays seeking medical assistance for some reason after perceiving physical abnormalities or discomfort, which is composed of pre-hospital delay (PHD) and in-hospital delay. PHD can be further divided into decision delay and transportation delay (8). Decision delay refers to the time period between the identification of uncomfortable symptoms and the decision to seek medical help. Transportation delay refers to the time period between the decision to seek medical help and arrival at the hospital (8). Measures such as optimizing clinical pathways, setting up chest pain centers and improving first-aid models can reduce in-hospital delay. With the continuous shortening of in-hospital delay, the impact of PHD on the prognosis of patients is particularly critical. The promotion of seamless first-aid mode and the improvement of emergency medical transportation have improved the transportation delay (9), so the longest stage of current delay is the patient's decision delay (10).

Determining the factors affecting decision time (DT) helps to shorten DT. Previous studies focused on the entire PHD and ignored the important contribution of decision delay (11, 12). Studies have pointed out that DT is essentially influenced by patients' knowledge attitudes and practice (13, 14). Health literacy (HL) refers to “the degree to which individuals have the capacity to obtain, process, and understand basic health information and services needed to make appropriate health decisions” (15). Patients with low HL are more likely to have poor understanding of disease, and have problems in understanding health care information and following disease management guidelines (16), resulting in prolonged DT. In addition to the differences in knowledge and skills, attitudes and motivation are also involved. Patients with inadequate HL are less willing to follow complex nursing plans, leading to poor prognosis (17). Decision delay as the most easily improved part of pre-hospital deserves our attention (18). At present, the impact of HL on decision delay is not clear, so the purpose of this study is to analyze the effect of HL on decision delay in Chinese patients with AMI, and to provide information for improving the survival rate and prognosis of AMI patients.

## Methods

This cross-sectional study used a continuous inclusion method to investigate all eligible AMI patients in the cardiology department of a hospital in Chongqing from October 3, 2020 to March 3, 2021. The inclusion criteria were: (1) be diagnosed with AMI, (2) voluntary participation in this study, (3) a clear mind with a certain ability to understand and speak, and able to complete the survey. Finally, a total of 217 patients participated in this study. We also performed *post-hoc* power analysis using G^*^power 3.1.9.7 (Kiel University, Kiel, Germany) software to test the power of our study. The power (1-β) was determined to be 0.99, based on logistic regression test, error probability (significance level) of 0.05, odds ratio (OR) of 9.32 and total sample size of 217.

### Instrument

The research invited relevant experts to collaborate on a specially designed questionnaire, and we improved it with a pre-survey. The questionnaire was a voluntary and anonymous survey. No private information and sensitive language. Before the investigation, we obtained the ethical approval from The Ethics Committee of Chongqing Medical University. Although there is no informed consent form that needs to be signed separately, there is a description of informed consent at the beginning of the questionnaire and respondents were informed that submission of the questionnaire implied informed consent. The final questionnaire consisted of four sections: (1) sociodemographic characteristics, (2) HL, (3) symptoms onset information, and (4) clinical information.

HL was evaluated with the Brief Health Literacy Screen (BHLS) that included three items: (1) “How confident are you filling out medical forms by yourself?” (2) “How often do you have someone (like a family member, friend, hospital or clinic worker, or caregiver) help you read hospital materials?” (3) “How often do you have problems learning about your medical condition because of difficulty understanding written information?” Each item was scored on a 5-point Likert scale, with a total score of 15. The higher the total score, the higher the HL level. Inadequate HL was defined as a total score ≤ 9. BHLS has been shown to be a valid measure of HL in clinical settings (19, 20). The same cutoff value has been used in other studies measuring HL in patients with heart disease (21, 22).

Symptom onset information included the location of the onset, whether there were family members around at the time of the onset, and the choice of patients within 20 min after the onset, and patients' self-reported DT, etc. According to the recommendations of the American Heart Association, a decision time of 60 min or more was considered a decision delay. Similar time cutoff values were used in other studies (23).

Clinical information was obtained by consulting the hospital's electronic medical record system. Myocardial enzymes used the results of the first admission examination. For some indicators recorded in the use range, in order to avoid data loss, we took the closest value for analysis. For example, myoglobin (Myo) >500 ng/mL, we took Myo = 501 for analysis.

### Data Analysis

Data was examined for their distribution characteristics. Continuous variables conformed to the normal distribution were reported as mean ± standard deviation and analyzed using the independent sample *T*-test, while those not conformed were reported as the median and inter-quartile range (IQR) and were analyzed using Mann Whitney *U*-test. Categorical variables were reported as frequencies and percentages and analyzed using the chi-square test. Binary logistic regression was used to analyze the relationship between HL and DT. The relative risk for longer DT was presented as OR with 95% confidence interval (95% CI). In order to obtain better performance parameters of the model, we selected variables into the final model by least absolute shrinkage and selection operator (LASSO), and determine whether specific confounding variables should retained based on the method of OR change. That is, remove each covariate on a one-at-a-time basis and determine whether the OR of HL variable has substantially changed (more than 10%). If eliminating such a variable results in no change in the odds ratio, then it is not needed to control for confounding. LASSO can select the independent variables that have a greater impact on the dependent variables and calculate the corresponding regression coefficients, and finally get a relatively simplified model. Finally, six variables were included and all variables were entered into the regression model simultaneously. Among them, HL total score is independent variable, decision delay is dependent variable, and others are covariates. All analyses were performed with SPSS version 20.0 (SPSS, Inc., Chicago, IL, USA). The significance was set at *P* < 0.05 and all statistical tests were two-tailed.

## Results

### Basic Information

A total of 217 AMI patients were enrolled in the study, including 51 (23.5%) women and 166 (76.5%) men. Age ranged from 30 to 85 years, most patients were over 45 years old, with the median age of 68 years (IQR = 57–75). 187 (86.2%) patients were married, and only 47 (21.7%) people had a college degree or above. The majority of patients lived in urban areas (89.9%), used smartphones (72.8%), did not live alone (91.2%) and had no relatives or friends of the doctor (71.0%). More details were shown in Table 1. One hundred thirty-five patients reported DT of 60 min or above, and the prevalence of decision delay was 62.2%. The median DT for the whole sample was 3 h (IQR = 0.5–23.5). The median DT for delayed group was 13 h (IQR = 4–50), which was more than 0.5 h (IQR = 0.2–0.7) of the non-delayed group. The comparison results of patients grouped by DT were also shown in Table 1.

**Table 1:** Characteristics of the study population and logistic regression analysis of the factors influencing decision delay.

**Variables**	**Total (*n* = 217)**	**Decision time**		**Statistics**	***P***	**Logistics model**	
		** <60 min (*n* = 82, 37.8%)**	**≥60 min (*n* = 135, 62.2%)**			**OR (95%CI)**	***P***
Gender				0.56	0.453		
Female	51 (23.5)	17 (20.7)	34 (25.2)				
Male	166 (76.5)	65 (79.3)	101 (74.8)				
Age (year)	68.0 (57.0, 75.0)	68.0 (53.5, 73.5)	67.0 (58.0, 75.0)	5,206.50	0.464		
Marital status				0.02	0.891		
Married	187 (86.2)	71 (86.6)	116 (85.9)				
Others	30 (13.8)	11 (13.4)	19 (14.1)				
Education				0.46	0.794		
≤ Primary school	72 (33.2)	25 (30.5)	47 (34.8)				
Middle school and high school	98 (45.2)	39 (47.6)	59 (43.7)				
≥College	47 (21.7)	18 (22.0)	29 (21.5)				
Residency				2.36	0.124		
Rural	22 (10.1)	5 (6.1)	17 (12.6)			1	
Urban	195 (89.9)	77 (93.9)	118 (87.4)			0.34 (0.08, 1.53)	0.160
Use smartphone				1.83	0.177		
No	59 (27.2)	18 (22.0)	41 (30.4)				
Yes	158 (72.8)	64 (78.0)	94 (69.6)				
Live with others				0.17	0.685		
Alone	19 (8.8)	8 (9.8)	11 (8.1)				
With family/roommates	198 (91.2)	74 (90.2)	124 (91.9)				
Doctor friends/relatives				0.14	0.713		
No	154 (71.0)	57 (69.5)	97 (71.9)			1	
Yes	63 (29.0)	25 (30.5)	38 (28.1)			4.54 (1.48, 13.95)	**0.008**
Heath literacy							
Adequate	59 (27.3)	39 (47.6)	20 (14.9)	27.29	** <0.000**	1	** <0.000**
Inadequate	157 (72.7)	43 (52.4)	114 (85.1)			9.32 (3.38, 25.70)	
Missing	1		1				
Symptom-onset-location				0.04	0.840		
Public place	41 (18.9)	15 (18.3)	26 (19.3)				
Home	175 (80.6)	67 (81.7)	108 (80.0)				
Missing	1		1				
Symptom-onset-situation				0.66	0.416		
Physical activities	68 (31.3)	23 (28.0)	45 (33.3)				
Rest	149 (68.7)	59 (72.0)	90 (66.7)				
Family around				2.08	0.150		
No	71 (32.7)	22 (26.8)	49 (36.3)				
Yes	146 (67.3)	60 (73.2)	86 (63.7)				
Responses to symptoms				78.30	** <0.000**		
Rest	61 (28.1)	17 (20.7)	44 (32.6)			1	
Handle it yourself	84 (38.7)	13 (15.9)	71 (52.6)			1.72 (0.67, 4.395)	0.259
Tell the people around	34 (15.7)	15 (18.3)	19 (14.1)			0.29 (0.10, 0.84)	**0.022**
Direct contact to hospital	38 (17.5)	37 (45.1)	1 (0.7)			0.01 (0.00, 0.03)	** <0.000**
BMI				1.68	0.196		
<25.0 kg/m^2^	133 (61.3)	46 (56.1)	87 (64.4)			1	
≥25 kg/m^2^	83 (38.2)	36 (43.9)	47 (34.8)			0.45 (0.20, 1.01)	**0.053**
Missing	1		1				
Symptoms persist				0.32	0.572		
No	96 (44.2)	34 (41.5)	62 (45.9)				
Yes	120 (55.3)	47 (57.3)	73 (54.1)				
Missing	1	1					
History of CAD				0.03	0.855		
No	184 (84.8)	70 (85.4)	114 (84.4)				
Yes	33 (15.2)	12 (14.6)	21 (15.6)				
Hypertension				1.63	0.201		
No	94 (43.3)	31 (37.8)	63 (46.7)				
Yes	123 (56.7)	51 (62.2)	72 (53.3)				
Diabetes				0.10	0.748		
No	132 (60.8)	51 (62.2)	81 (60.0)				
Yes	85 (39.2)	31 (37.8)	54 (40.0)				
Combined with HF				0.36	0.546		
No	181 (83.4)	70 (85.4)	111 (82.2)				
Yes	36 (16.6)	12 (14.6)	24 (17.8)				
Current smoker				0.13	0.716		
No	104 (47.9)	38 (46.3)	66 (48.9)				
Yes	113 (52.1)	44 (53.7)	69 (51.1)				
Current drinker				0.19	0.667		
No	139 (64.1)	54 (65.9)	85 (63.0)				
Yes	78 (35.9)	28 (34.1)	50 (37.0)				
CK-MB (ng/mL)	10.8 (2.7, 63.2)	12.5 (3.3, 71.8)	8 (2.5, 58.5)	4,811.00	0.279		
Missing	5	2	3				
cTnI (ng/mL)	1.0 (0.2, 5.8)	1.2 (0.2, 6.1)	1 (0.2, 4.8)	5,096.50	0.568		
Missing	4	1	3				
Myo (ng/mL)	129.0 (44.7, 501.0)	244.5 (63.4, 501.0)	105.0 (40.2, 501.0)	4,474.50	0.074		
Missing	6	2	4				
NT-proBNP (pg/ml)	508.5 (118.0, 2,055.8)	189.0 (76.4, 1,113.5)	720.0 (185.0, 2,373.5)	3,913.50	**0.001**		
Missing	3	1	2				
Diagnosis				3.73	0.053		
NSTEML	70 (32.3)	20 (24.4)	50 (37.0)			1	
STEML	147 (67.7)	62 (75.6)	85 (63.0)			0.20 (0.07, 0.59)	**0.004**

*Bold means P <0.05*.

*BMI, body mass index; CAD, coronary artery disease; CK-MB, creatine kinase-MB; cTnI, cardiac troponin-I; HF, heart failure; Myo, myoglobin; NSTEML, non-ST elevation myocardial infarction; NT-proBNP, N-terminal pro B type natriuretic peptide; STEML, ST-elevation myocardial infarction*.

### Symptom Onset Information

More than half of the patients had symptoms at home (80.6%), at rest (68.7%), with family members around (67.3%). In order to explore the causes of decision delay, we specially set a question to ask patients what action they took within 20 min of discovering symptoms. Interestingly, more people in the non-delayed group chose to go directly to the hospital (45.1%) while most people in the delayed group chose to handle it by themselves (52.6%) (*P* < 0.000). More details were shown in Table 1.

### Clinical Information

Among 217 AMI patients, 56.7% had hypertension, 39.2% had diabetes, 16.6% had HF, only 15.2% had a history of CAD. The past medical history must be confirmed by the diagnosis of the hospital, not by the self-report of the patients. Among the four myocardial indexes collected, creatine kinase-MB (CK-MB), cardiac troponin-I (cTnI), and Myo of the non-delayed group were higher than those of the delayed group, only N-terminal pro B type natriuretic peptide (NT-proBNP) was lower than that in the delayed group (*P* = 0.001). See Table 1 for more details.

### HL

There were 39 (47.6%) patients with adequate HL in the non-delayed group, while only 20 (14.9%) patients in the delayed group (*P* < 0.000). We compared the three items separately so as to make the difference in HL between the two groups clearer, the results showed that the score of each item in the non-delayed group was higher than that of the delayed group (*P* < 0.000). In general, patients with shorter DT had better HL. See Table 2 for more details.

**Table 2:** Comparison of HL between different decision time.

**Variables**	**Decision time**		***P***
	** <60 min (*n* = 82)**	**≥60 min (*n* = 134)**	
HL1	2.73 ± 1.08	2.01 ± 0.96	** <0.000**
HL2	3.67 ± 0.79	2.81 ± 0.96	** <0.000**
HL3	2.82 ± 1.11	2.19 ± 0.95	** <0.000**
HL	9.22 ± 2.38	7.02 ± 2.38	** <0.000**

*Bold means P <0.05*.

*HL, health literacy*.

## Discussion

Few studies have focused on the effect of HL on decision delay in AMI patients, and most of them measuring the entire PHD (11, 12). As far as we know, the current measurement of PHD is not consistent (8), “the period from symptom onset to at the hospital” is dominant, but many studies have not given a clear definition of symptom onset. In our study, the decision delay was defined as “the time period between the identification of uncomfortable symptoms and decision to seek medical help,” which further reduced the error and improved the accuracy, because some symptoms were not immediately recognized by patients. We believe that the time from the onset of symptoms to the recognition of patients cannot be counted as part of DT, because the patient does not realize they need to make a decision at this time.

The median time of decision delay in 217 patients in this study was 180 min, slightly higher than that of another survey of 250 AMI patients in Shanghai, China (24). There were differences in the measurement results of DT for AMI patients in different countries and regions. A multicenter study involving 12 countries including the UK, France, Germany, etc. showed that DT of Finnish patients were shortest (median DT was 40 min) and that of the UK was the longest (median DT was 74 min) (25). The Global Registry of Acute Coronary Events project showed that patients from Australia/New Zealand had the shortest PHD (median PHD was 120 min in STEML patients and 150 min in NSTEML patients) and those from Argentina and Brazil had the longest delays (median PHD was 186 min in STEML patients and 240 min in NSTEML patients) (26). The “golden time” after the onset of AMI is 60 min (27). Before that, reperfusion therapy can effectively limit the infarct size and protect left ventricular function. The current status of PHD is still worrisome. Time is life for patients. How to promote patients to receive medical treatment as soon as possible is a great challenge for health care workers around the world.

After adjusting sociodemographic and clinical variables, inadequate HL still increased the risk of decision delay (OR = 9.32, *P* < 0.000). HL is a complex concept that involves many aspects of individual skills, including reading, calculation, critical thinking, information acquisition, decision making and many other individual and social skills. These skills are essential in the self-management of diseases (28). Many studies have shown that patients' knowledge and behavior are important factors affecting decision delay (29–31). About one-third of patients did not have typical chest pain symptoms when they sought medical attention (32). Patients with poor awareness of symptoms can easily underestimate the severity of their disease and delay seeking treatment. In our results, we found that NSTEML patients have longer DT, which may be related to the fact that these patients often experience atypical symptoms (33). Longer DT is also associated with low patient numeracy (34), possibly because numeracy skills help patients evaluate risk and integrate available information more precisely into decision-making models (35). Decision delay is the main cause of PHD, and it is also the only part that can be improved by intervention (18). Therefore, shortening DT is an effective way to improve the survival rate of patients, and improving HL is a potentially effective strategy. Improving HL is a gradual, lifelong process and interventions should be integrated into daily life. Organized and systematic health education should be carried out in schools, communities, hospitals and other places to spread health knowledge and popular science of atypical symptoms, improve people's knowledge reserves, and make decisions quickly when detecting symptoms (36).

## Limitations

Several limitations of this study should be considered. Participants were limited to hospitals in Chongqing, China, which may lead to selection bias and results with limited generalizability. Data was obtained based on hospital medical records and patients' self-reports, which may be affected by recall bias. Although the cutoff value of 9 points is currently the most commonly used in standard, it is not necessarily the best choice. There is no unified scoring standard for BHLS, some use 0–4 points and some use 1–5 points. At the same time, the optimal cutoff value of screening tools in different settings depends on tools accuracy, prevalence of inadequate HL, the cost of false positive classification, and the benefits of identifying true positives.

## Conclusion

When examined in a binary logistic regression model that includes patient behavior, sociodemographic variables, and clinical characteristics, inadequate HL in AMI patients was a key predictor of decision delay. Patients with BMI <25 kg/m^2^, inadequate HL, self-treatment after onset, and diagnosed with NSTEML and with doctor's relatives or friends were more likely to have longer DT.

## Data Availability Statement

The original contributions presented in the study are included in the article/supplementary material, further inquiries can be directed to the corresponding author.

## Ethics Statement

The studies involving human participants were reviewed and approved the Ethical Review Committee (IRB) at Chongqing Medical University, Chongqing, China. Written informed consent for participation was not required for this study in accordance with the national legislation and the institutional requirements.

## Author Contributions

Z-yF, YY, and FZ conceived the study. R-yY and LT prepared the study materials. Z-yF wrote the original draft and FZ gave the entire process technical and paper writing guidance support. All authors have read and approved the final manuscript.

## Conflict of Interest

The authors declare that the research was conducted in the absence of any commercial or financial relationships that could be construed as a potential conflict of interest.

## Publisher's Note

All claims expressed in this article are solely those of the authors and do not necessarily represent those of their affiliated organizations, or those of the publisher, the editors and the reviewers. Any product that may be evaluated in this article, or claim that may be made by its manufacturer, is not guaranteed or endorsed by the publisher.
